# The importance of plant growth-promoting rhizobacteria to increase air pollution tolerance index (APTI) in the plants of green belt to control dust hazards

**DOI:** 10.3389/fpls.2023.1098368

**Published:** 2023-03-10

**Authors:** Mahmood Najafi Zilaie, Asghar Mosleh Arani, Hassan Etesami

**Affiliations:** ^1^ Department of Environmental Sciences, Faculty of Natural Resources, Yazd University, Yazd, Iran; ^2^ Department of Soil Science, University of Tehran, Tehran, Iran

**Keywords:** *Bacillus pumilus*, *Haloxylon aphyllum*, *Nitraria schoberi*, plant growth-promoting rhizobacteria, *Seidlitzia Rosmarinus*, *Zhihengliuella halotolerans*

## Abstract

Dust causes adverse effects on the physiological and biochemical characteristics of plants and limits their use in the development of the green belt. Air Pollution Tolerance Index (APTI) is an important tool to screen out plants, based on their tolerance or sensitivity level to different air pollutants. The aim of this study was to investigate the effect of two plant growth-promoting bacterial strains (*Zhihengliuella halotolerans* SB and *Bacillus pumilus* HR) and their combination as a biological solution on APTI of three desert plant species of *Seidlitzia rosmarinus*, *Haloxylon aphyllum* and *Nitraria schoberi* under dust stress (0 and 1.5 g m^-2^ 30 days^-1^). Dust caused a significant decrease of 21% and 19%, respectively, in the total chlorophyll of *N. schoberi* and *S. rosmarinus*, an 8% decrease in leaf relative water content, a 7% decrease in the APTI of *N. schoberi*, and a decrease of 26 and 17% in protein content of *H. aphyllum* and *N. schoberi*, respectively. However, *Z. halotolerans* SB increased the amount of total chlorophyll in *H. aphyllum* and *S. rosmarinus* by 236% and 21%, respectively, and the amount of ascorbic acid by 75% and 67% in *H. aphyllum* and *N. schoberi*, respectively. *B. pumilus* HR also increased the leaf relative water content in *H. aphyllum* and *N. schoberi* by 10% and 15%, respectively. The inoculation with *B. pumilus* HR, *Z. halotolerans* SB and the combination of these two isolates decreased the activity of peroxidase by 70%, 51%, and 36%, respectively, in *N. schoberi*, and 62%, 89%, and 25% in *S. rosmarinus*, respectively. These bacterial strains also increased the concentration of protein in all three desert plants. Under dust stress, *H. aphyllum* had a higher APTI than the other two species. *Z. halotolerans* SB, which had been isolated from *S. rosmarinus*, was more effective than *B. pumilus* HR in alleviating the effects of dust stress on this plant. Therefore, it was concluded that plant growth-promoting rhizobacteria can be effective at improving the mechanisms of plant tolerance to air pollution in the green belt.

## 1 Introduction

Dust is a collection of fine particles of natural or industrial origin that is considered as one of the most widespread air pollutants. In recent years, the presence of dust particles suspended in the air has become a major environmental problem (Abbasi et al., 2019). Although dust pollution has always been a natural phenomenon, its amount sometimes reaches an unacceptable level, affecting all aspects of human life. In recent years, human activities have intensified the phenomenon (Middleton, 2019).

Plants can naturally absorb fine dust particles from the atmosphere and act as an air cleaner filter. The development of green plants around the cities is one of the most appropriate biological methods to reduce air pollution in these areas (Alotaibi et al., 2020). Since urban green belts can largely prevent the entry of fine dust and pollution into cities, their creation has gained special importance (Hamraz et al., 2014). Nowadays the use of plants resistance to environmental stress, such as dust, in creating green spaces is very important and a priority. The importance of these plants can be understood by the fact that they not only purify the air by biofiltering pollutants through absorption, impingement and adsorption (Escobedo et al., 2008), but also have positive effects on the soil and water quality of the area, in addition to adding aesthetic value to it (Pradhan et al., 2016). Leaves are prominent receivers of air pollutants, but show variable behavior towards different types of air pollutants (Sahu and Sahu, 2015). A few plants show tolerance to a particular air pollutant, while others have high mortality rates and are therefore sensitive (Nadgórska–Socha et al., 2017). Tolerant species are suitable for planting, while sensitive species can be used as both bioindicators and biomonitors to provide quantitative and qualitative information, respectively, about the surrounding environment (Priyanka and Dibyendu, 2009; Trivedy and Goel, 2010; Kwon et al., 2020).

It has been reported that the gaseous and particulate pollutants in the atmosphere have direct effect on many morphological, physiological and biochemical characteristics of plants (Steubing et al., 1989) such as chlorophyll *a* and *b*, photosynthesis, the metabolism of proline, the activity of antioxidant enzymes such as catalase and peroxidase, leaf relative water content, ascorbic acid concentration, protein concentration, soluble sugars and pH (Najafi Zilaie et al., 2022a; Najafi Zilaie et al., 2022b; Zilaie et al., 2022). Although the tolerance of plants to these pollutants has been studied according to different aspects such as peroxidative activity (Xu et al., 2014), leaf conductance, glutathione concentration (Pasqualini et al., 2001; Hoque et al., 2007), membrane permeability (Farooq and Beg, 1980) and other biochemical parameters, contradictory results have been obtained when the parameters are studied individually even for the same species. Accordingly, Singh and Rao (1983) developed a formula through the aggregation of four biochemical parameters (relative water content, ascorbic acid, chlorophyll content, and leaf extract pH), known as Air Pollution Tolerance Index (APTI). The APTI has been praised by a wide range of researchers for its combination of different parameters that show more reliable results than any analysis based on a single parameter (Neel et al., 2018; Bellini and De Tullio, 2019; Manjunath and Reddy, 2019).

Plant growth-promoting rhizobacteria (PGPR) have been shown to improve the plant tolerance to environmental stresses by various mechanisms (Etesami and Maheshwari, 2018). These bacteria have multiple plant growth-promoting (PGP) traits under different stressful conditions (Etesami and Maheshwari, 2018; Etesami and Glick, 2020). In the study of Tiepo et al. (2018), the use of PGPR (*Azospirillum brasilense* and *Bacillus* sp.) improved the physiological traits of *Cariniana estrellensis* seedlings such as relative water content, water potential, intracellular CO_2_ concentration and electrolyte leakage under water deficit stress conditions. In another study, the inoculation of mesquite transplants with *Azospirillum brasilense* showed a positive effect on the morphological and physiological parameters of the plant (e.g., biomass, root system, and chlorophyll *a* and *b*) under drought stress conditions. Hajiabadi et al. (2021) also isolated two bacterial isolates of *B. pumilus* and *Z. halotolerans*, from the rhizosphere of *Halostachys belangeriana* and *S. rosmarinus*, respectively, and measured their PGP traits. The results of these researchers indicated that the mentioned bacterial isolates have several PGP traits such as the ability to solubilize phosphates, produce 1-aminocyclopropane-1-carboxylic acid (ACC) deaminase enzyme, auxin, and siderophore and fix N_2_. The inoculation of these two bacterial isolates also improved the tolerance of wheat to salinity and increased its growth and yield. The positive effect of *B. pumilus* (Ansari et al., 2019) and *Z. halotolerans* (Jha et al., 2012) on plant growth has also been reported in other studies. In the present study, the effect of single inoculation and co-inoculation of these two bacterial isolates *Bacillus pumilus* HR, isolated from *Halostachys belangeriana*, and *Z. halotolerans* SB, isolated from *S. rosmarinus*, on the APTI of three desert plant species of *S. rosmarinus*, *H. aphyllum* and *N. schoberi* was investigated under dust stress. The research questions of this study were: (*i*) Would these bacterial strains improve APTI of three desert plant species of *S. rosmarinus*, *H. aphyllum* and *N. schoberi*; (*ii*) Would the bacterial strain isolated from the rhizosphere of *S. rosmarinus* have a greater effect on the tolerance of this plant against pollution stress compared to the bacterial strain isolated from another desert plant; and (*iii*) Would the combination of these two bacterial strains have a greater effect on plant growth compared to their single inoculation. To our knowledge, this is the first study to investigate the effect of PGPR on APTI of plants. Therefore, the results of this study are promising and can be used for better management of planting desert plants of the green belt around cities in dry areas.

## 2 Materials and methods

### 2.1 Rhizobacterial strains and inoculum preparation

In this study, two rhizobacterial strains, *B. pumilus* HR (accession number: MW295357), previously isolated from the halophyte *Halostachys belangeriana*, and *Z. halotolerans* SB (accession number: MW295355), previously isolated from the halophyte *S. rosmarinus*, were used (Hajiabadi et al., 2021). These bacterial strains were characterized in our previous study (Hajiabadi et al., 2021). These strains were also able to colonize the roots of *S*. *rosmarinus* (Zilaie et al., 2022)*, H. aphyllum* (Najafi Zilaie et al., 2022b), and *N. schoberi* (Najafi Zilaie et al., 2022a) under salinity and dust stress conditions. For preparing the bacterial inoculum, two bacterial strains were grown in a nutrient broth (NB) culture medium separately until they both reached the late exponential-phase (3 × 10^8^ cells mL^−1^) at 28 ± 2°C. The bacterial cells were then washed twice and re-suspended in sterile distilled water. Since the two bacterial strains were supposed to be inoculated into these three plants together, we also showed that the two strains did not have any antagonistic effects on each other’s growth by an *in vitro* assay (Najafi Zilaie et al., 2022b).

### 2.2 Treatments and experimental design

To evaluate the effect of two strains HR and SB on APTI of three desert plant species (*S. rosmarinus*, *H. aphyllum* and *N. schoberi*) under dust stress, a plant growth assay was carried out in a completely randomized design with factorial arrangement (4 × 2 × 3) with three replications in a research greenhouse located at Department of Environmental Engineering, Yazd-University, Yazd, Iran. The experimental treatments included: (a) rhizobacterial strain factor at four levels: (i) non-inoculated seedlings (control), (ii) seedlings inoculated with *B. pumilus* HR, (iii) seedlings inoculated with *Z. halotolerans* SB, and (iv) seedlings co-inoculated with the HR and SB strains; (b) dust stress factor at 2 levels: 0 and 1.5 g m^-2^ 30 days^-1^; and (c) plant species factor (*S. rosmarinus*, *H. aphyllum* and *N. schoberi*).

The non–sterile soil (3 kg) used in plastic pots (40 cm × 25 cm) with drainage holes was air-dried at room temperature and passed through a 4–mm sieve. Soil properties are as follows: pH, 7.61; soil texture, sandy clay loam (28.2% clay, 12% silt, and 59.2% sand); Na, 2.15 meq kg^−1^; organic matter (OM), 1.8 g kg^−1^; ${\text{SO}}_{4}^{2-}$ , 48 meq kg^−1^; electrical conductivity (EC), 2.0 dS m^−1^; total nitrogen, 0.20 g kg^−1^; Ca, 1.8 meq kg^−1^; available phosphorus, 15.0 mg kg^−1^; NH_4_OAc–K, 368 mg kg^−1^; and calcium carbonate (CaCO_3_) equivalent, 32.5%. The properties of dust used are as follows: sand, 22.24%; clay, 21.83%; gravel, 2.01%; silt, 43.74%; calcium, 22.70%; magnesium, 2.42%; iron, 4.71%; sodium, 2.37%; aluminium, 4.08%; potassium, 0.79; zinc, 52.90 mg kg^-1^; cupper, 15.80 mg kg^-1^; cobalt, 12.70 mg kg^-1^; uranium, 1.96 mg kg^-1^; cadmium, 0.60 mg kg^-1^; nickel, 94.00 mg kg^-1^; vanadium, 73.40 mg kg^-1^; barium, 231.00 mg kg^-1^; chromium, 116 mg kg^-1^; and lead, 20.23 mg kg^-1^.

One-year seedlings of the same size of *S. rosmarinus*, *H. aphyllum* and *N. schoberi* were obtained from the nursery of Natural Resources and Watershed Management General Office of Yazd Province, Iran. The roots of these seedlings were immersed in each bacterial suspension (3 × 10^8^ CFU mL^−1^) for two hours or in sterile water as un-inoculated control at room temperature (25°C). The *S. rosmarinus*, *H. aphyllum* and *N. schoberi* seedlings were also co-inoculated with both *Z. halotolerans* SB and *B. pumilus* HR by being immersed in equal volumes of the suspension of these two bacteria. After inoculation, the seedlings were singularly transferred to pots. Dust treatments were applied by dust simulator (Dustin-mizer Model 1212, https://www.amazon.com/Dustin-mizer-Model-1212-Includes-Deflector/dp/B002KIB680) 30 days after culturing these seedlings in the pots. The dust treatment was applied at 1.5 g m^-2^ 30 days^-1^ once a week for 150 days according to a previous assay (Ahmadi Foroushani et al., 2021). After pouring the dust into the valve of the dust simulator, the content of dust (g m^-2^) was controlled by using a trap with dimensions of 1.6 × 2.3 m^2^. When applying dust, all the control plants were moved outside the greenhouse so that dust does not settle on them. The plants were grown in a glasshouse at 25 ± 2°C with a photoperiod of 16-h light and 8-h darkness and 60% relative humidity. At the end of the experiment period (150 days after planting in pots), APTI, peroxidase activity, and protein concentration were measured.

### 2.3 Measurements

#### 2.3.1 APTI determination

Air pollution tolerance index (APTI) was determined according to the following formula (Zouari et al., 2018):


APTI=[A(T+P)+R]/10

Where A, T, P, and R are ascorbic acid, total chlorophyll, pH of leaf extract and leaf relative water content, respectively.

#### 2.3.2 Ascorbic acid determination

Ascorbic acid content of young and completely expanded leaves (one sample from each plant) was determined based on the spectrophotometric method according to a previous study (Mukherjee and Choudhuri, 1983). Briefly, the leaf sample (100 mg) was extracted with 10 mL of 1% metaphosphoric acid for 45 min at room temperature and filtered through Whatman No. 4 filter paper. The filtrate (1 mL) was then mixed with 9 mL of 2,6-dichlorophenolindophenol, and the absorbance was measured within 30 min at 520 nm against a blank. The content of ascorbic acid was calculated on the basis of the calibration curve of authentic L-ascorbic acid. The content of ascorbic acid was expressed in mg g^−1^ FW (fresh weight) of tissue.

#### 2.3.3 Total chlorophyll determination

To extract total chlorophyll (chlorophyll *a* and chlorophyll *b*), 0.5 g fresh leaf samples (young and completely expanded leaves) were taken and homogenized with 10 mL of 80% acetone. The homogenized samples were centrifuged at 4,000 rpm for 10 min at 4°C. The supernatants were separated from the mixture and collected in a cuvette for further use. To measure chlorophyll *a* and chlorophyll *b*, the spectrophotometric method proposed by Lightenthaler (1987) was employed using a UV-VIS spectrophotometer (Varian Cary 50; Varian GmbH, Darmstadt, Germany) to measure absorbance of the extracts at 663.2 nm and 646.8 nm. The concentrations of the photosynthetic pigments present in the extracts were estimated using the following equation:


$$\text{Total chlorophyll=}\left[12.5\left({\text{A}}_{663.2}\right)-2.79\left({\text{A}}_{646.8}\right)\right]\;+\left[21.5\left({\text{A}}_{646.8}\right)-5.1\left({\text{A}}_{663.2}\right)\right]$$

#### 2.3.4 Leaf extract pH determination

Leaf extract pH was determined according to the protocol of Tak and Kakde (2017). For each treatment, four samples (0.5 g) of fresh leaves (young and completely expanded leaves) were crushed and homogenized in 50 mL deionized water, after which the mixture was centrifuged at 7,000×g for 10 min. The pH of the supernatant was measured using a digital pH meter (EYELA, Japan).

#### 2.3.5 Relative water content determination

For determination of relative water content (RWC), each leaf (young and completely expanded leaves) was weighed on a digital scale (GF-300; A&D Company, Tokyo, Japan) with an accuracy of 0.001 g (FW) and then placed in Falcon tubes completely filled with distilled water. The tubes were left in the dark at 4°C for 24 h. After this period, the leaves were removed from water and placed on absorbent paper to remove excess water, and turgid weight (TW) was determined. Afterward, the leaves were dried at 70°C until stabilization, and dry weight was determined (DW). Leaf RWC was calculated using the following equation and expressed as a percentage (Ritchie et al., 1990):


RWC(%)=[(FW-DW)/(TW-DW)]×100

#### 2.3.6 Peroxidase activity determination

To measure the peroxidase (EC1.11.1.7) activity of young and completely expanded leaves, extraction from fresh leaves (0.5 g) was performed according to Mukherjee and Choudhuri (1983). The extract was frozen in liquid nitrogen and then ground in phosphate buffer (100 mM, pH 7.0). Homogenates were centrifuged at 4°C for 10 min under 15,000×g. The supernatant was kept at 4°C until used to measure the activity of peroxidase. The method of Hemeda and Klein (1990) was used to evaluate peroxidase activity. The rate of guaiacol oxidation in the presence of H_2_O_2_ recorded at 470 nm specifies the enzyme activity (U mg^-1^ protein).

#### 2.3.7 Protein concentration determination

Protein concentration was estimated according to the method described by Bradford (1976). Young and completely expanded leaves (25 mg) were homogenized with 1 mL of TRIS-HCl (0.1 M) buffer and then 20 μL supernatant was mixed with 80 μL of distilled water. Then, 900 μL Bradford reagent was added and incubated for 2 min. The absorbance was measured at 595 nm in an UV–VIS spectrophotometer (Varian Cary 50; Varian GmbH, Darmstadt, Germany). The concentration of protein was determined using bovine serum albumin as standard. The concentration of protein was expressed as microgram bovine serum albumin (BSA) equivalent (μg BSA)/g leaves fresh weight.

### 2.4 Statistical analysis of data

After confirming the normality of the data by Kolmogorov–Smirnov test, experimental data were analyzed by three-way analysis of variance (ANOVA) using the SAS v.9.1 (SAS Institute Inc., Cary, NC) statistical analysis software. *Post-hoc* comparisons of means (mean ± *SE*, *n = 3*) using Tukey’s multiple-range test at *p*<0.05 were used to compare significant difference among treatments.

## 3 Results

### 3.1 Effect of strains on total chlorophyll of dust-stressed plant species

**Figure 1:**
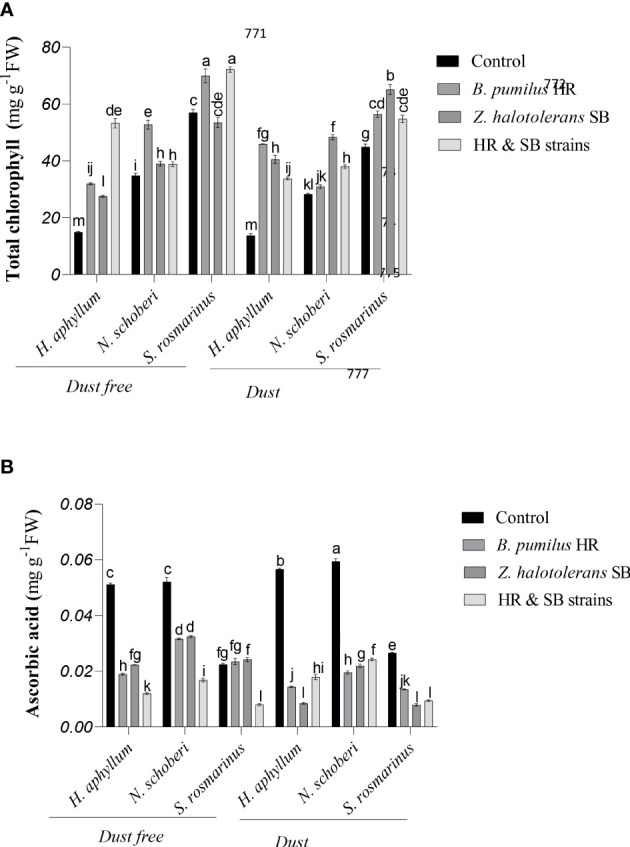
Triple interactions of treatments of bacterial strains, three desert plant species of *S. rosmarinus*, *H. aphyllum* and *N. schoberi* and dust on the content of total chlorophyll **(A)** and ascorbic acid **(B)** of the leaves of three plant *species* grown under greenhouse conditions for five months. Alphabets indicate significant differences among various treatments (Means ± SE, n = 3) according to Tukey’s multiple-range test at *p*<0.05.

Dust treatment alone (bacteria free) decreased the amount of total chlorophyll by 18% and 22% in *N. schoberi* and *S. rosmarinus*, respectively (**Figure 1A**). In dust free plants, the combination of two isolates (HR and SB isolates) caused a significant increase of 256% and 27% in total chlorophyll of *H. aphyllum* and *S. rosmarinus* plants, respectively, while in *N. schoberi* plant, *B. pumilus* HR strain caused the highest increase (52%) compared to its control. Under dust conditions, HR isolate increased the total amount of chlorophyll in *H. aphyllum* plant (236%), while in *N. schoberi* and *S. rosmarinus* plants, *Z. halotolerans* SB strain increased the amount of total chlorophyll compared to its control (respectively, 71% and 44%). The results also indicated that the effect of bacterial inoculation on increasing total chlorophyll was higher in *H. aphyllum* plant than the other two species (**Figure 1A**).

### 3.2 Effect of strains on ascorbic acid of dust-stressed plant species

In *H. aphyllum*, *N. schoberi* and *S. rosmarinus* plants, the application of dust in the absence of bacteria caused a significant increase of 11%, 14% and 19% in ascorbic acid of these plants, respectively (**Figure 1B**). In dust free plants, the combination of two isolates significantly decreased ascorbic acid by 77%, 69% and 66%, respectively, in *H. aphyllum*, *N. schoberi* and *S. rosmarinus* compared to the control. Under dust conditions, SB isolate caused the highest decrease in ascorbic acid amount in *H. aphyllum* and *S. rosmarinus* plant (respectively, -85% and -70%), while in *N. schoberi* plants, HR strain caused the highest decrease in ascorbic acid amount compared to its control (-67%). The results also showed that the effect of bacterial inoculation on decreasing ascorbic acid was higher in *H. aphyllum* plant than the other two species (**Figure 1B**).

### 3.3 Effect of strains on leaf pH of dust-stressed plant species

**Figure 2:**
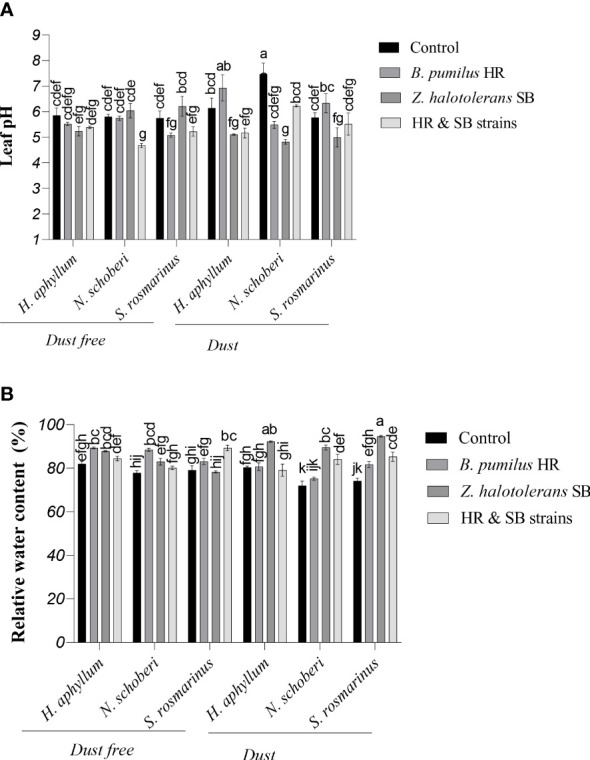
Triple interactions of treatments of bacterial strains, three desert plant species of *S. rosmarinus*, *H. aphyllum* and *N. schoberi* and dust on leaf pH **(A)** and relative water content **(B)** of the leaves of three plant *species* grown under greenhouse conditions for five months. Alphabets indicate significant differences among various treatments (Means ± SE, n = 3) according to Tukey’s multiple-range test at *p*<0.05.

In *N. schoberi* plant, the use of dust treatment in the absence of bacteria caused a 29% increase in leaf pH (**Figure 2A**). In dust free *H. aphyllum* plant, the inoculation with a combination of two isolates decreased the leaf pH by 19% compared to the control. Under dust conditions, in all three plants of *H. aphyllum*, *N. schoberi* and *S. rosmarinus*, SB isolate caused the greatest decrease in leaf pH compared to its control (-17%, -35% and -13%, respectively). The results also showed that the effect of bacterial inoculation on decreasing leaf pH was higher in *N. schoberi* plant than the other two species (**Figure 2A**).

### 3.4 Effect of strains on RWC of dust-stressed plant species

Under dust-free conditions, the inoculation with HR strain in *H. aphyllum* and *N. schoberi* plants increased the amount of relative water content **(**RWC) by 9% and 14% compared to the control, while the combination of two isolates in *S. rosmarinus* increased RWC by 12% compared to the control (**Figure 2B**). Under dust conditions in all three plants *H. aphyllum*, *N. schoberi* and *S. rosmarinus*, the inoculation with SB isolate caused a significant increase in RWC by 15%, 24% and 28%, respectively, compared to the control. The results also indicated that the effect of bacterial inoculation on increasing RWC was higher in *S. rosmarinus* plant than the other two species (**Figure 2B**).

### 3.5 Effect of strains on APTI of dust-stressed plant species

**Figure 3:**
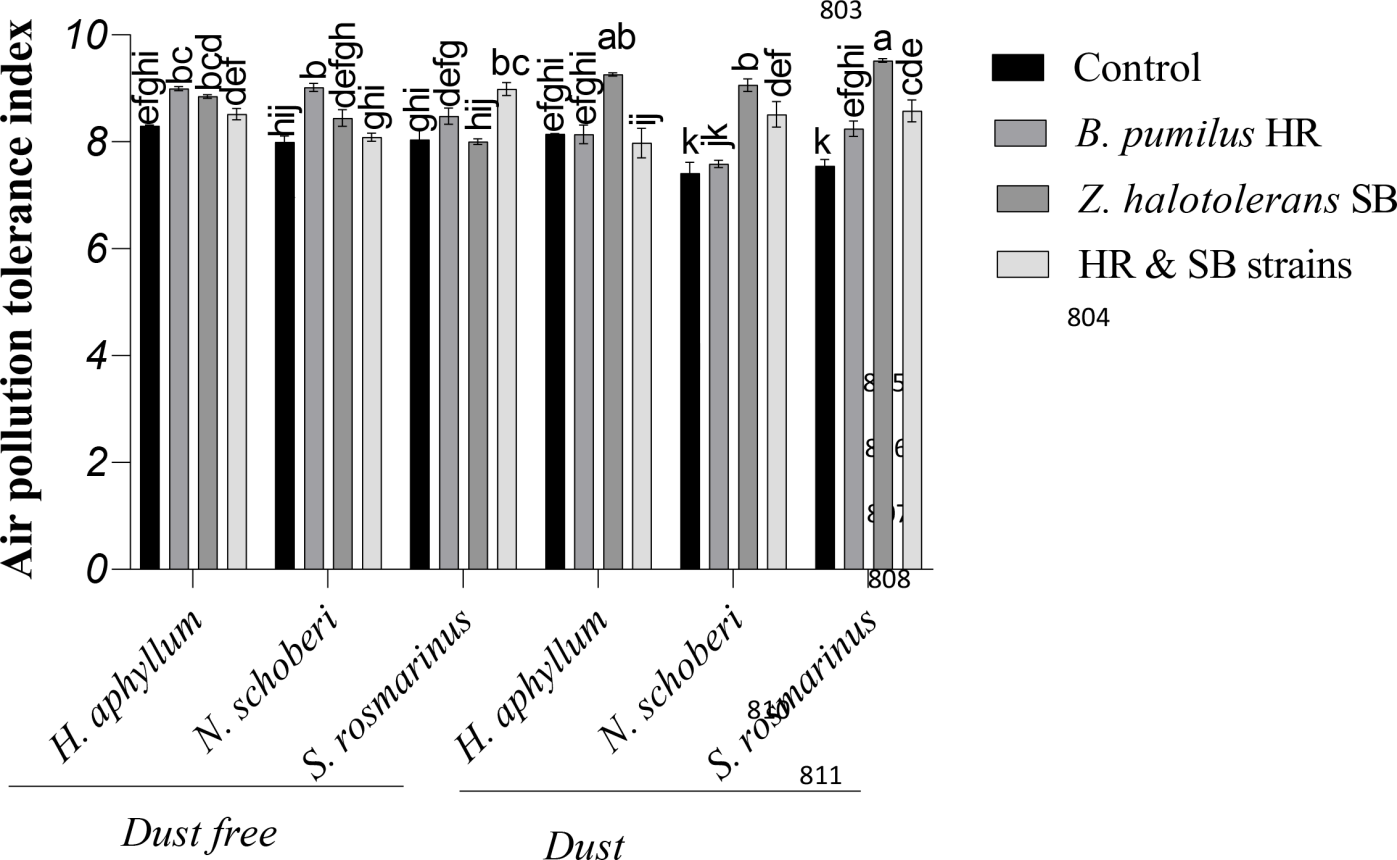
Triple interactions of treatments of bacterial strains, three desert plant species of *S. rosmarinus*, *H. aphyllum* and *N. schoberi* and dust on air pollution tolerance index (APTI) of three plant *species* grown under greenhouse conditions for five months. Alphabets indicate significant differences among various treatments (Means ± SE, n = 3) according to Tukey’s multiple-range test at *p*<0.05.

Under the dust free conditions, HR strain caused the most significant increase of APTI by 8% and 13%, respectively, in *H. aphyllum* and *N. schoberi* plants compared to control, while in *S. rosmarinus* plant, the co-inoculation of two isolates increased APTI by 11%. Under dust conditions, SB isolate caused the greatest increase in APTI in *H. aphyllum*, *N. schoberi* and *S. rosmarinus* plants compared to their controls (14%, 22% and 26%, respectively). The results also showed that the effect of bacterial inoculation on increasing APTI was higher in *S. rosmarinus* plant than the other two species (**Figure 3**).

### 3.6 Effect of strains on peroxidase of dust-stressed plant species

**Figure 4:**
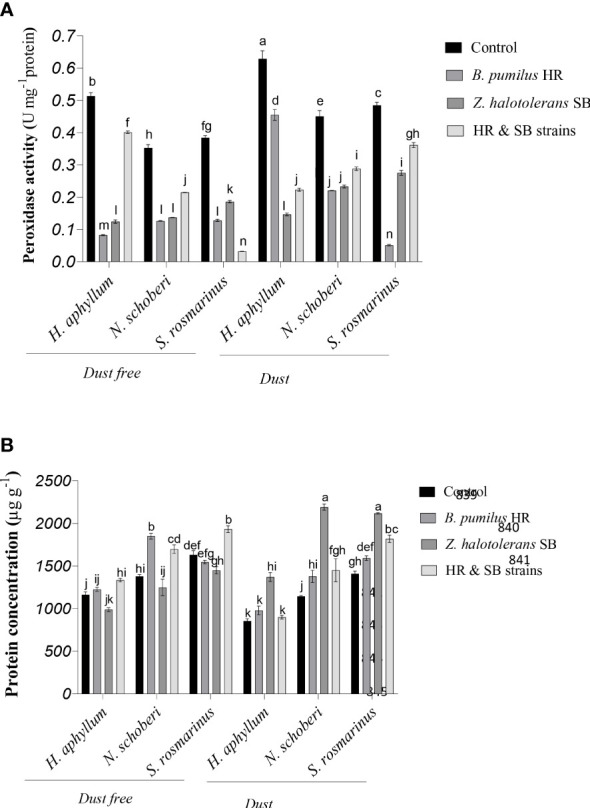
Triple interactions of treatments of bacterial strains, three desert plant species of *S. rosmarinus*, *H. aphyllum* and *N. schoberi* and dust on peroxidase activity **(A)** and protein concentration **(B)** of the leaves of three plant *species* grown under greenhouse conditions for five months. Alphabets indicate significant differences among various treatments (Means ± SE, n = 3) according to Tukey’s multiple-range test at *p*<0.05.

In *H. aphyllum*, *N. schoberi* and *S. rosmarinus* plants, the application of dust treatment in the absence of bacteria caused a significant increase of 23%, 28% and 26% in peroxidase, respectively (**Figure 4A**). In dust free plants, the inoculation with HR isolate, SB isolate and the combination of these two isolates decreased the activity of peroxidase by 84%, 76% and 22% respectively, in *H. aphyllum* plant, by 64%, 60% and 39% in *N. schoberi* plant, and by 67%, 52% and 88% in *S. rosmarinus*, respectively, compared to the control. Also, under dust conditions, HR isolate, SB isolate and the combination of two isolates reduced peroxidase activity by 28%, 77% and 64% in *H. aphyllum* plant, by 51%, 48% and 36% in *N. schoberi* plant and by 90%, 43% and 25% in *S. rosmarinus* plant, respectively, compared to the control. The results also indicated that the effect of bacterial inoculation on increasing peroxidase activity was higher in *S. rosmarinus* plant than the other two species (**Figure 4A**).

### 3.7 Effect of strains on protein of dust-stressed plant species

In *H. aphyllum*, *N. schoberi* and *S. rosmarinus* plants, the application of dust in the absence of bacteria significantly decreased protein concentration by 26%, 17%, and 14%, respectively (**Figure 4B**). In dust free plants, the inoculation of bacteria caused a significant increase in the concentration of protein, as the combination of two isolates increased protein concentration by 15% and 18% in *H. aphyllum* and *S. rosmarinus*, respectively, compared to the control. In *N. schoberi* plant, the inoculation with HR isolate and the combination of two isolates increased the concentration of protein by 34% and 23%, respectively, compared to the control. Also, under dust conditions, the inoculation with bacteria caused a significant increase in the concentration of protein, as in the inoculation of SB strain, the concentration of protein increased by 60%, 91% and 50%, respectively, in *H. aphyllum*, *N. schoberi* and *S. rosmarinus* plants compared to the control. The results also indicated that the effect of bacterial inoculation on increasing protein concentration was higher in *N. schoberi* plant than the other two species (**Figure 4B**).

## 4 Discussion

Dust causes adverse effects on the physiological and biochemical characteristics of plants (Sharifi et al., 2019; Meravi et al., 2021; Yaghmaei et al., 2022) and limits their use in the development of the green belt. In the present study, we also showed that dust could negatively affect the characteristics of three desert plant species of *S. rosmarinus*, *H. aphyllum* and *N. schoberi*. In the present research, with the application of dust, the amount of total chlorophyll in *N. schoberi* and *S. rosmarinus* species decreased significantly compared to the control, but this decrease was not significant in *H. aphyllum* species. An increase in dust concentration also induced a significant decrease in the content of total chlorophyll in four plant species (*Fraxinus rotundifolia, Morus alba, Colias caucasica*, and *Melia azedarach*) (Javanmard et al., 2019). A similar trend of reduced total chlorophyll in response to dust pollution has also been reported on *H. aphyllum* Bunge (Heydarnezhad, 2014; Najafi Zilaie et al., 2022b) and *S. rosmarinus* (Zilaie et al., 2022). Dust deposition on leaf surface may decrease the synthesis of chlorophyll because of shading effects (Sarma et al., 2017; Setsungnern et al., 2018). Another reason may be related to pigment degradation and inhibition of enzymes essential for biosynthesis of pigments due to incorporation of dust particles into leaf tissues (Lepeduš et al., 2003). In the present study, the application of bacterial strains increased the concentration of total chlorophyll in all three species. Under dust conditions, the effect of dust particle application on plant physiology is similar to the effects of drought stress (Rai, 2016). The increase in the amount of total chlorophyll in the plants inoculated with beneficial bacteria under dust conditions in the present study may be due to the ability of the bacterial strains to produce siderophore and auxin, as well as their ability to increase the absorption of nutrients involved in the structure of chlorophyll such as Mn and Mg (Zilaie et al., 2022; Najafi Zilaie et al., 2022a). Siderophores have a strong tendency to absorb iron (III), which is an essential nutrient for chlorophyll construction (Santoyo et al., 2019). The results of the present study also showed that the highest concentration of total chlorophyll was registered in *H. aphyllum* plant inoculated with *Z. halotolerans*; this can be due to the significant effect of two PGP traits of siderophore and auxin hormone in this bacterium (Hajiabadi et al., 2021).

Ascorbic acid in the leaves of plants has multiple functions through cell wall synthesis, cell division, photosynthetic carbon fixation, and acts as a strong reducing agent to protect plants against reactive oxygen species (ROS); thus it improves the ability of plants to withstand air pollution (Sahu et al., 2020). The level of tolerance in plants increases with increasing ascorbic acid content (Lima et al., 2000). The findings of the presented research also indicated that the amount of ascorbic acid in the leaves of three species increased significantly with the application of dust. This indicates that the three plant species respond to oxidative stress caused by dust with an increase in ascorbic acid content. These results are in line with previous research findings (Meerabai et al., 2012; Sahu et al., 2020). Leaf pH is an index of detoxification mechanism in plants for improving tolerance capacity against air pollution (Ninave et al., 2001). The leaf extract pH is known to impact the ascorbic acid synthesis of plants (Singare and Talpade, 2013). Increase in the leaf extract pH value has been reported to efficiently convert the hexose sugar to ascorbic acid and therefore a high pH is considered good for the tolerance of plants against air pollution (Shannigrahi et al., 2004; Enete and Ogbonna, 2012). The findings of the presented research also indicated that the leaf extract pH in *N. schoberi* species increased significantly with the application of dust. The penetration of chemical dust particles with an alkaline nature into the cell sap and their conversion to radicals cause an increase in leaf extract pH. High leaf pH also increases the efficiency of conversion from hexose to ascorbic acid (Singare and Talpade, 2013). In the current study, it seems likely that the penetration of alkaline dust particles (pH 7.6) into the leaf tissues, along with growing dust accumulation on the leaf surface, caused increased pH in the leaf extracts. In the present study, the bacterial strains significantly decreased the concentration of leaf ascorbic acid and pH of the leaf extract under dust stress, among which the SB strain had the most effect. Since plant oxidative stress is reduced by bacterial inoculation, the stress response leading to ascorbate production is less intense. Decreased ascorbic acid concentration in the plants inoculated with PGPB under dust stress has been reported in other studies (Walia et al., 2019; Najafi Zilaie et al., 2022b). These bacteria seemingly improved plant tolerance, but decreased plant tolerance responses.

Water content in plant leaves help maintain the physiological water balance in adverse environmental conditions (Yadav and Pandey, 2020). High relative water content of leaf favours transpiration rate and pollution resistance in plants (Jyothi and Jaya, 2010). The results of the presented research indicated that dust caused a significant decrease in RWC in *N. schoberi* species, but this decrease was not significant in *H. aphyllum* and *S. rosmarinus* species. This result is in line with earlier studies on other plants (Maletsika et al., 2015; Karami et al., 2017). The lowest relative water content among the investigated plant species was related to *N. schoberi* species, whose relative water content was equal to 71.95%. In a similar research, the application of dust treatment led to a decrease in the relative water content (Javanmard et al., 2019). Low RWC may be due to the effect of dust pollutant on leaf transpiration, since the crust formed by dust deposition on leaves can lead to blockage of the stomata and reduced transpiration (Zia-Khan et al., 2014; Masoud et al., 2019). Also, the reaction of dust particles with the cell membrane results in foliar injury and higher membrane permeability in dusted leaves, which may be another reason for low RWC value (Yaghmaei et al., 2022). Dust particles also cause loss of water and soluble nutrients in plants by increasing the permeability of plant cells and eventually causing premature aging of leaves (Meravi et al., 2021). Therefore, plants with a RWC less affected by stress were more resistant to dust (Taheri Analojeh et al., 2016). The results of the presented study showed that the inoculation of three plants with PGPR strains increased the relative water content in these plant species. The highest leaf RWC was observed in the plants inoculated with two strains (*Z. halotolerans* SB + *B. pumilus* HR) followed by the plants inoculated with *B. pumilus* HR strain compared to control crops under dust stress. One of the reasons for this increase in the plant species inoculated with bacterial strains could be the production of auxin and, thus, increase in the length and weight of roots compared to the control plant species under stress conditions (Etesami and Maheshwari, 2018; Najafi Zilaie et al., 2022b). These bacterial strains may also decrease the production of abscisic acid (Naz et al., 2009); in this way, they decrease the negative effects of stress on stomatal conductance, photosynthesis, and plant sensitivity to water deficiency (Etesami and Maheshwari, 2018). It seems that the plants inoculated with the bacterial strains have the ability to change the structure of the lateral root system and increase RWC (Bertrand et al., 2015; Shirmohammadi et al., 2020). Increased leaf RWC under stress in the plants inoculated with PGPR has also been reported in other studies (Tahir et al., 2017; Cedeño-García et al., 2018; Zilaie et al., 2022). In general, an increase in the number of lateral roots and root hairs causes addition of root surfaces available for nutrient and water uptake. Higher water and nutrient uptake by inoculated roots cause an improved water status of plants, which in turn could be the main factor enhancing plant growth (Etesami and Maheshwari, 2018).

The application of dust resulted in a significant decrease in APTI in *N. schoberi* species, but this decrease was not significant in *H. aphyllum* and *S. rosmarinus* species. The APTI indicates the plant’s ability to tolerate air pollution (Molnár et al., 2020). The reduction of APTI under dust conditions in the present research can be due to the effect of dust particles on the parameters of APTI. When the plant absorbs dust particles on the surface of its leaves, adverse effects occur at the physiological (change in pH and loss of relative water content) and biochemical (decrease in chlorophyll concentration) levels, which causes a decrease in APTI. APTI reduction under dust stress has been determined in other studies (Chaudhary and Rathore, 2019; Yadav and Pandey, 2020). The inoculation of these three plants with PGPR strains under dust stress increased APTI in the plants, as the inoculation with *B. pumilus* HR isolate had the greatest effect on increasing APTI in *N. schoberi* species. It can be assumed that the use of PGPR leads to the improvement of photosynthesis, and finally increasing the plant’s tolerance to dust stress by increasing the water use efficiency and relative water content as well as the concentration of chlorophyll (through the production of siderophore and auxin) and reducing pH. The results of the present study also showed that *H. aphyllum* species had a higher APTI (the least affected by stress) than the other two species. The plant species having high APTI are supposed to have greater defence against air pollution. This corroborates the earlier reports that the plants exposed to polluted environment tend to increase their tolerance ability with high APTI. Whereas the plants showing a decrease in APTI can be used as indicator (Prajapati and Tripathi, 2008; Jyothi and Jaya, 2010). The idea can also be used to identify the plants suited for plantation.

The results of the present study also showed that with the application of dust, the peroxidase activity of these plants increased. The highest and the lowest activity of leaf peroxidase enzyme were related to the non-inoculated *H. aphyllum* under dust stress (0.63 U mg^-1^ protein) and *B. pumilus* HR-inoculated *H. aphyllum* (0.12 U mg^-1^ protein), respectively. The closing of the stomata by dust particles in the plant tissue is the fastest reaction of plants in response to the presence of dust, which disrupts the photosynthesis process, increases ROS and creates oxidative stress in the plants. The increase in the active forms of oxygen under stress increases the activity of some antioxidant enzymes such as peroxidase, which ultimately leads to the plant’s tolerance to environmental stresses (Ghanem et al., 2021). In the study of Najafi Zilaie et al. (2022b), the activity of peroxidase enzyme also increased in plants under dust stress. In the present study, the inoculation with PGPR strains decreased the peroxidase enzyme activity in these plants, among which *B. pumilus* HR isolate had the greatest effect on reducing the peroxidase activity of leaves. It can be supposed that PGPR reduced the concentration of ROS and free radicals by creating suitable conditions for the plant growth, and as a result, the activity of the crop’s antioxidant defense systems decreased. PGPR participate in reducing stress by increasing water absorption through producing auxin and enhancing root volume, which justifies the reduction in the production of antioxidants (Etesami and Maheshwari, 2018).

In this study, the amount of protein decreased under dust stress. It seems that the decrease in the photosynthetic efficiency of plants under dust stress has caused a decrease in the accumulation of photosynthetic materials, which has also led to a decrease in protein concentration (Setsungnern et al., 2018). In addition, due to the closing of the stomata, the production of free radicals (Thompson et al., 1984) may have played a role in damaging the protein structure. According to Treesubsuntorn et al. (2021), a decrease in protein production occurs under dust stress. In the present study, the inoculation of plants with bacterial isolates under dust stress increased the protein concentration in the plants, as the highest increase in the concentration of protein (67.83 µg g^-1^) among the studied species was observed in the *N. schoberi* species inoculated with the combination of two bacteria isolates. Preventing the destruction of protein or stimulating their synthesis with the support of biological fixation of N_2_ is one of the strategies of bacterial strains to withstand the stress of the crop, which ultimately causes an increase in the concentration of protein in the plant (Nawaz et al., 2020). Previously, nitrogen fixation by the same genera of studied bacteria has been reported (Emami et al., 2019). An increase in protein production under stressful conditions following the treatment of plant with PGPR has also been reported in other studies (Liu et al., 2019; Pan et al., 2019).

*H. aphyllum* species had the highest APTI compared to the other two species, and bacterial strains increased this index in the studied species. As mentioned before, one of the reasons for increasing RWC in plants inoculated with PGPR can be the production of growth phytohormones and the development of the root system to improve the absorption and efficiency of water use under stress conditions (Baldan et al., 2015). The plants inoculated with PGPR have the ability to alter the structure of the lateral root system and increase water absorption. It is known that PGPR decrease the negative effects of stress on plants by producing abscisic acid and thereby reduce the plant’s sensitivity to water shortage to some extent (Etesami and Beattie, 2017). The increase of water inside the plant tissue (as one of the determining parameters of APTI) increases the APTI in the plant. The results of the present research also indicated that bacterial strains especially SB strain caused a further increase in the relative water content in *H. aphyllum* species.

## 5 Conclusions

The results of the research indicated that the physiological parameters of three important desert plants (*S. rosmarinus*, *H. aphyllum* and *N. schoberi*) were negatively affected by dust stress. However, PGPR (Z*. halotolerans* SB and *B. pumilus* HR) could attenuate the adverse effects of the dust stress on these plants. Bacterial strain SB isolated from *S. rosmarinus* had a greater effect on alleviating the negative impacts of dust on most of the indicators measured in this plant than *B. pumilus* HR. However, *B. pumilus* HR performed better on *H. aphyllum* and *N. schoberi*, which explains the importance of bacteria-plant interaction. The results also showed that *H. aphyllum* species had a higher air pollution tolerance index (APTI) than the other two species. It was also concluded that the application of PGPR can improve the tolerance of these plants to air pollution (APTI) by improving the effective parameters. Therefore, in order to increase the tolerance of green belt plants to air pollution, the use of PGPR should also be considered as an effective factor in such studies.

## Data availability statement

The original contributions presented in the study are included in the article/supplementary materials. Further inquiries can be directed to the corresponding author.

## Author contributions

All authors contributed to the article and approved the submitted version.
